# Direct Analysis of Psilocin and Muscimol in Urine Samples Using Single Drop Microextraction Technique In-Line with Capillary Electrophoresis

**DOI:** 10.3390/molecules25071566

**Published:** 2020-03-29

**Authors:** Anna Poliwoda, Katarzyna Zielińska, Piotr P. Wieczorek

**Affiliations:** Faculty of Chemistry, University of Opole, Oleska 48, 45-042 Opole, Poland,

**Keywords:** psilocin, muscimol, single drop microextraction, capillary electrophoresis, urine, green chemistry

## Abstract

The fully automated system of single drop microextraction coupled with capillary electrophoresis (SDME-CE) was developed for in-line preconcentration and determination of muscimol (MUS) and psilocin (PSC) from urine samples. Those two analytes are characteristic active metabolites of *Amanita* and *Psilocybe* mushrooms, evoking visual and auditory hallucinations. Study analytes were selectively extracted from the donor phase (urine samples, pH 4) into the organic phase (a drop of octanol layer), and re-extracted to the acidic acceptor (background electrolyte, BGE), consisting of 25 mM phosphate buffer (pH 3). The optimized conditions for the extraction procedure of a 200 µL urine sample allowed us to obtain more than a 170-fold enrichment effect. The calibration curves were linear in the range of 0.05–50 mg L^−1^, with the correlation coefficients from 0.9911 to 0.9992. The limit of detections was determined by spiking blank urine samples with appropriate standards, i.e., 0.004 mg L^−1^ for PSC and 0.016 mg L^−1^ for MUS, respectively. The limits of quantification varied from 0.014 mg L^−1^ for PSC and 0.045 mg L^−1^ for MUS. The developed method practically eliminated the sample clean-up step, which was limited only to simple dilution (1:1, *v*/*v*) and pH adjustment.

## 1. Introduction

Some species of mushrooms (especially *Amanita* and *Psilocybe*) are sources of naturally occurring psychedelic substances. The best known hallucinogenic ingredients are ibotenic acid (IBO), muscimol (MUS), psilocybin (PSB), and psilocin. The main psychoactive constituents in *Amanita* species are ibotenic acid (IBO) and muscimol [1,2]. The psychoactive dose of muscimol is estimated to be in the range of 10–15 mg, whereas that of ibotenic acid is 50–100 mg [3]. Generally, 100 g of dried *Amanita muscaria* contains about 150 mg of MUS and 30 mg of IBO; therefore, 10 g of dried mushrooms can evoke a psychedelic effect. The toxicity of MUS–LD_50_ values in mice is 2.5 mg/kg [4]. Their psychotropic properties result from their ability to activate the *N*-methyl-d-aspartate glutamate and GABA receptors [5].

The components of *Psilocybe* are indole hallucinogenic alkaloids. These mushrooms grow in Middle and North Europe, both Americas, Asia, Australia, and Oceania [2]. The common mushroom, *Psilocybe cubensis*, typically contains 1–2 mg psilocin/g dried mushroom. The hallucinogenic effect of psilocin is evoked at doses in the range from 8 to 10 mg [6]. According to the literature data, the LD_50_ value of PSC in mice is 290 mg/kg [7]. In contrast to MUS and IBO, these indole alkaloids have a similar chemical structure to the human neurotransmitter serotonin, and behave as serotonin receptors agonists [6,8,9].

The risks associated with the consumption of these mushrooms result not only from their toxicity, but also from the fact that they possess ambivalent potential. This means that they can evoke either favorable or adverse reactions to living organisms. Therefore, the measurement of those analytes, both in mushroom extracts and body fluids matrices, is essential in quantitative and qualitative analyses to investigate and monitor poisoning status. For this reason, it is significant to have a reliable and fast analytical method for diagnostic examination of intoxication with hallucinogenic mushrooms. At present, only a few analytical procedures exist, including the LC-MS/MS [10], GC-MS [11], or CE-MS [12] techniques, which were developed to determine muscimol and ibotenic acid in serum or urine samples. Generally, the available literature focuses upon the analysis of mushroom extracts in which the content of analyzed analytes is significantly higher [13,14,15,16]. A few more papers describe the methods of psilocybin and psilocin analysis in physiological fluids, the most popular of which are those using HPLC with a mass spectrometry detector (MS) [17,18,19,20,21,22,23] and photodiode array detector (PDA) [24], or using electrochemical detection [9,25]. Some analytical procedures with GC-MS [26,27] and CE-UV [28] have also been used. However, a sample pretreatment step for sample clean-up and analyte preconcentration is often required.

Consequently, in this work, we focused on two fungal hallucinogenic metabolites: muscimol and psilocin. These two compounds provide definite proof of hallucinogenic mushroom poisoning. To our knowledge, no analytical methods have been developed to simultaneously detect muscimol and psilocin in biological samples (urine). The method described in this work consists of a fully automated system, i.e., single drop microextraction technique in-line with capillary electrophoresis (SDME-CE) without any sample clean-up step. The proposed analytical procedure may be useful in forensic investigations.

Recently, the combination of a single drop microextraction technique in-line with capillary electrophoresis was applied for the analysis of selected organic compounds [29,30,31,32,33,34,35,36,37,38]. The main advantage of the SDME-CE technique is that the simultaneous isolation, preconcentration, and determination of various analytes is possible in a fully automated test. In this case, the sample components (analytes) are selectively extracted into a thin layer of organic solvent (extractant) that covers the drop of the acceptor phase suspended at the inlet end of the capillary. In the next step, analytes are re-extracted into the acceptor phase and analyzed in-line with a capillary electrophoresis method [39]. Due to the drop volume (a few nanoliters), the SDME technique is an analytical procedure that radically reduces the consumption of solvents. Furthermore, high enrichment factors that result in low detection limits can be easily obtained.

## 2. Results and Discussion

### 2.1. Optimization of SDME-CE parameters

For the optimization of the SDME-CE method, major factors, e.g., the volume of donor phase and its pH, the type of organic solvent used for drop formation, as well as the time of extraction, need to be investigated. Therefore, the first stage of this research included a procedure for optimizing the extraction conditions of aqueous solutions of standard substances (a mixture of MUS and PSC).

#### 2.1.1. Selection of the Organic Phase and Drop Formation

The applicability of SDME is limited to the appropriate choice of organic solvent to be used for drop formation. The applied solvent should have low density and low solubility in the aqueous donor and acceptor phases. A low density of organic solvent ensures the stability of a single drop. Additionally, it should have a good ability to extract analytes and high viscosity to form a well-settled phase. Therefore, nonpolar solvents (octanol, toluene, dichloromethane, and ethyl acetate), as well as their mixtures (1:1, *v*/*v*) in which studied compounds are soluble, were tested. For example, when toluene was used separately, both hallucinogens were extracted with very low extraction efficiency (below 10 %). For comparison, dichloromethane and ethyl acetate could be used, but as an ingredient of a mixture rather than as a single solvent, because of their volatility. Usually, working with these solvents causes problems with maintaining a stable drop hanging at the tip of the capillary.

**Figure 1:**
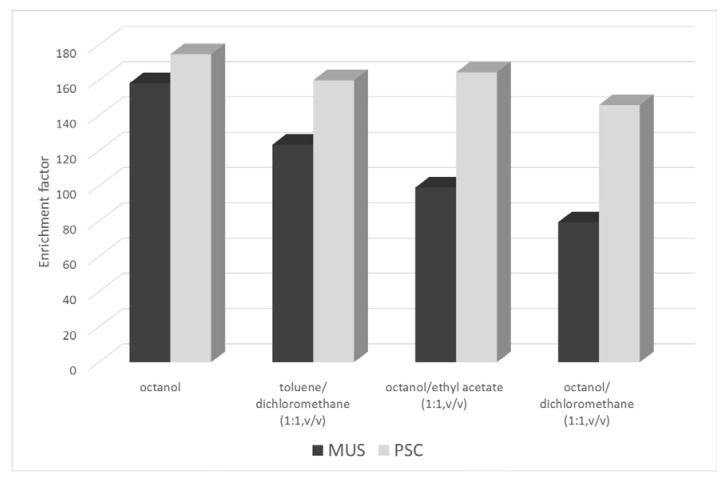
Effect of organic solvent on the enrichment factor of the studied analytes in aqueous samples using the SDME-CE system. Sample concentration: 25 mg L^−1^, pH 4; CE conditions: BGE—25 mM phosphate buffer pH 3; Sample volume: 200 µL; Organic phase: various organic solvents; extraction time: 180 s; injection: 0.1 psi, 1s; *n* = 6, RSD < 6.2%.

Finally, four studied solvent configurations (octanol, octanol with ethyl acetate, octanol with dichloromethane, and toluene with dichloromethane) allowed us to get the desired results (Figure 1). However, the best enrichment factor was observed when octanol was applied; therefore, it was used in further experiments. Moreover, octanol enabled us to obtain a stable organic surface (organic phase) that covered the drop of the acceptor phase suspended at the inlet end of the capillary. Undesirable phenomenon of drop dissolution with an extended extraction time were not present only with octanol.

#### 2.1.2. Adjustment of the Composition of Acceptor and Donor Phase

The most crucial parameter in SDME-CE is the choice of acceptor phase. The acceptor phase affects analyte extraction during single drop microextraction procedures, as well as the resolution and sensitivity of the analytes during CE separation. Therefore, the effect of several buffers with different concentrations and pHs was tested. An increase of the pH of the acceptor phase (CE buffer) led to high EOF, which increased the mobility of analytes, but also increased the deprotonation of the studied analytes and their low affinity to the organic phase (octanol). Furthermore, under these conditions, problems with peaks tailings and the Joule heating effect arose. It was found that 25 mM phosphate buffer (pH 3) used as a CE electrolyte yielded both efficient and reproducible electrophoretic resolution and enabled to obtain high enrichment factors. Furthermore, the organic phase volume is a critical parameter for the extraction efficiency and kinetics. By playing with back-pressure and its time, the influence of the n-octanol volume was investigated. Although the use of lower volumes of organic extractive phase led to higher extraction efficiency, the repeatability values were poor.

**Figure 2:**
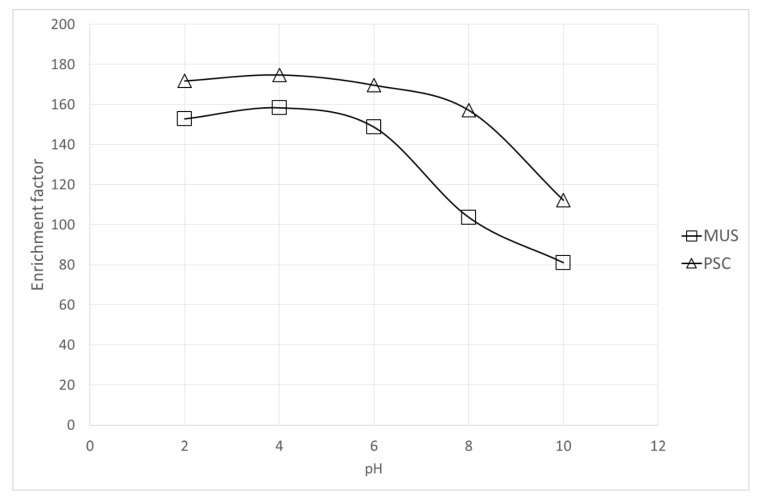
Preconcentration of MUS and PSC versus pH of the donor phase. Sample concentration: 25 mg L^−1^, at pH in range 2–8; CE conditions: BGE—25 mM phosphate buffer pH 3; Sample volume: 200 µL; Organic phase: octanol; extraction time: 180 s; injection: 0.1 psi, 1s; *n* = 6, RSD < 4.8%.

Another important parameter to obtain a high enrichment factor is the pH of the donor phase. In this case, the donor phase (aqueous samples) at different pH values (2; 3; 4; 6; 8; 10) was examined. Figure 2 demonstrates the effect of donor phase pH on the enrichment of MUS and PSC by the SDME in-line CE system. For both analytes, the best enrichment factor was obtained with an acidic pH. Generally, the increase of donor phase pH from 4 to 10 resulted in decreasing the enrichment factors of the studied analytes. Good recoveries were achieved at pH 4, so this was selected for further experiments.

The obtained results confirmed that in the case of the muscimol and psilocin, the acidic pH of the sample solution increased the preconcentration effect of the studied analytes. As for samples with pH ≤ 4, the obtained enrichment factor was the highest. This pH influenced the presence of analyte molecules in aqueous sample solution in a cationic form (MUS pKa 4.8 and 8.4, PSC pKa 9.4). At the same time, the cationic form of the studied analytes limited its direct extraction through the hydrophobic octanol phase that covered the drop of an acceptor phase during the SDME process. Therefore, presumably on the donor side, there must be a process that, despite the presence of positively charged muscimol and psilocin molecules, facilitates their diffusion into octanol. This factor is supposed to be orthophosphoric acid, which is present in the sample (as it was used for pH adjustment). Thus, taking into account the phosphoric acid pKa values (2.12; 7.21; and 12.67), at pH 4, the dominant form in the sample solution is negative ion H_2_PO_4_^−^ (almost 100% molecules). For example, this negatively charged counter ion (H_2_PO_4_^−^) is able to interact (forming an ion-pair) with a highly hydrophilic, positively charge amino group in muscimol. This phenomenon seems to decrease the overall muscimol hydrophilicity and increase the affinity of the neutral ion-pair for the hydrophobic phase of octanol. There is no direct proof of the existence of an ion pair between the studied analytes and phosphate ions. Presumably, by analogy of amino acids and peptide separations by reversed-phase liquid chromatography, the effect of anionic ion-pairing reagent interaction also influences the enrichment of the cationic form of analytes [40]. Furthermore, at the beginning of the project, 80% *w*/*w* acetic acid was used to acidify the samples (pKa 4.75). In this case, the desirable preconcentration effect was not observed, which may indirectly confirm the presence of an anionic ion-pairing reagent effect.

Furthermore, if the extraction proceeds as long as the concentration gradient is preserved, the enrichment factor will be 1, but only in the case when the equilibrium stage is observed, and the concentration of the form of studied analytes is the same in both the acceptor and donor phases. In the developed SDME method, the donor phase pH was 4, whereas acceptor phase pH was 3 (phosphate buffer). Such a small change in the pH value of the aqueous phases significantly influences the number of negatively charge H_2_PO_4_^−^ ions presented in the solutions. In the acceptor phase (pH 3), the number of H_2_PO_4_^−^ ions decreases (only about 75–80% of the total molecules, according to the dissociation diagram). Thus, fewer ion pairs are formed on the acceptor side, and therefore, an increase of positively charged analyte molecules is observed. Moreover, the presence of ionized particles prevents them from re-entering the organic phase of octanol. It is enough to observe concentration gradient (and thus the rate of mass transfer) of the diffusing species. Furthermore, on the basis of similarities with membrane extraction, the concentration difference, ΔC, over the organic phase, can be written as:

ΔC = α_D_C_D_ − α_A_C_A_

where C_D_ and C_A_ are the total concentrations in the donor (sample) and acceptor phases, respectively, and α_D_ and α_A_ are the fractions of the analytes that are in extractable (ion-pair) form in the indicated phase [41,42]. In studied SDME system, the α_D_ and α_A_ values seem to play a significant role; therefore, the enrichment of the studied analytes, observed at an acceptor phase of pH 3, was sufficient for the present purposes.

The effect of donor phase volume was examined too, as the volume of the donor phase results in the achievement of the equilibrium state. Thus, different values of sample volume (100, 200, 500, 1000, and 1400 μL) were tested. In each case, no significant differences between the obtained peak areas were observed. Therefore, a volume of 200 µL was selected as the optimal parameter. In that volume, chemical equilibrium was established, and more compounds were not extracted to the acceptor phase.

#### 2.1.3. Influence of the Extraction Time and Injection Parameters

Additionally, the effect of extraction time was examined to improve extraction efficiency. The time of extraction can increase the analyte equilibrium between two phases: organic and aqueous. Analytes can migrate from the donor phase to an organic solvent over a period. Furthermore, excessive extraction time can cause drop instability. In contrast, a short extraction time may cause problems with achieving an equilibrium state. The three-phase SDME-CE system is a combination of three simultaneously occurring processes: extraction of the compound from an aqueous donor phase, diffusion through a hydrophobic organic thin octanol layer, and re-extraction to the aqueous acceptor phase. By adjusting the pH of the donor and acceptor phases, SDME can be carried out effectively. It is known that the form of these analytes will change with the change of solution pH and thereby affect their water-solubility and extractability.

The influence of extraction time was evaluated by applying different times: 1, 3, 6, 9, and 12 min. Generally, extraction times longer than 3 minutes decreased extraction efficiency, and the peak areas of analytes were smaller. Above 6 minutes of extraction, the examined compounds were not preconcentrated, and the dissolution of the drop into the donor and the acceptor phase was observed. Therefore, for further analysis, we chose 3 minutes as the extraction time. 

**Figure 3:**
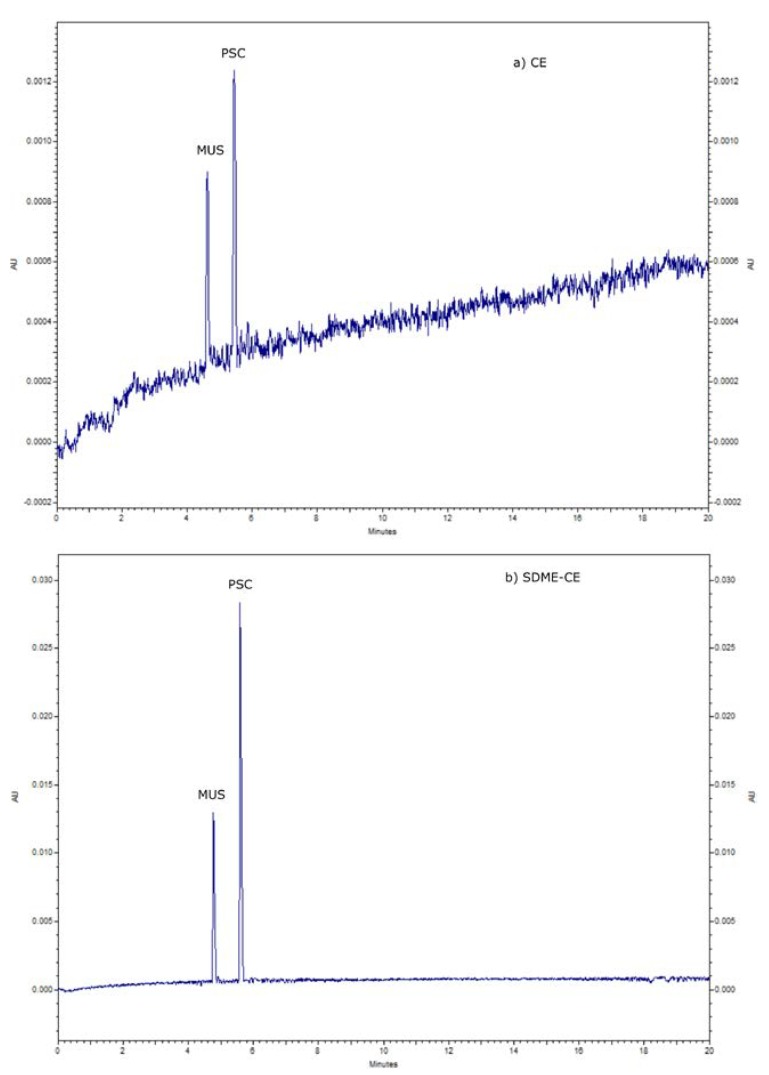
Electropherograms of 25 mg L^−1^ aqueous samples of standards (PSC and MUS) during CE analysis (**a**) and enriched by 3 min SDME-CE (**b**). CE conditions: BGE—25 mM phosphate buffer pH 3; SDME-CE conditions: Sample volume: 200 µL; Organic phase: octanol; extraction time: 180 s; injection: 0.1 psi, 1s; *n* = 6, RSD < 5.9%).

Injection time is another crucial factor in the SDME-CE procedure. Any addition of organic solvents into capillary electrophoresis can lead to problems with the current. It is essential to inject only the acceptor phase, without the organic solvent (octanol). In this case, a hydrodynamic injection was applied, and the best injection time was 1 s under a pressure of 0.1 psi. Finally, the best preconcentration effect and separation efficiency were obtained for the following conditions: donor phase pH—4, donor phase volume—200 µL, extraction time—3 min, injection—1 s at a pressure of 0.1 psi, and octanol used as an organic phase. Figure 3 shows the electropherogram obtained during the analysis of an aqueous mixture of standards PSC and MUS at a concentration of 25 mg L^−1^, with (a) and without (b) the preconcentration step using developed SDME in-line CE method. The presented results indicate that the analyzed compounds were sufficiently preconcentrated and separated using the developed SDME-CE system. The total time of analysis was less than 7 minutes. The obtained enrichment factors were 158 for MUS and 174 for PSC, respectively. Recovery values of over 89% for muscimol and almost 97 % for psilocin were obtained, taking into consideration that the maximum amount of enrichment factor for this SDME-CE working system was about 178.

### 2.2. Real Samples Analysis

**Figure 4:**
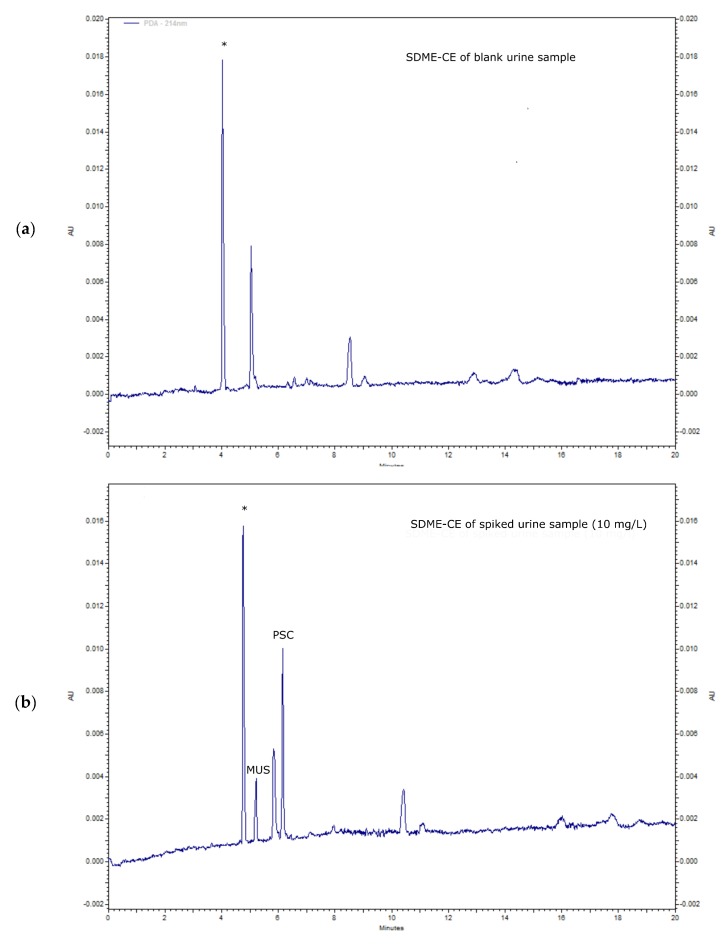
SDME-CE analysis of urine samples: electropherograms of (**a**) blank urine and (**b**) spiked urine sample with PSC and MUS (10 mg L^−1^). SDME-CE conditions: BGE—25 mM phosphate buffer pH 3 (also acceptor phase); Sample volume: 200 µL of blank urine or spiked urine; Organic phase (single drop): octanol; Extraction time: 180 s; Injection: 0.1 psi, 1s; *n* = 6, RSD < 8.5%).* -creatinine.

The applicability of the developed SDME-CE method was demonstrated for the urine sample analysis. The obtained electropherograms from a SDME-CE analysis of a blank urine sample (a) and a sample spiked with 10 mg L^−1^ of the studied hallucinogenic metabolites are presented in Figure 4. This clearly shows the sample clean-up capability of SDME, as well as the sample preconcentration effect. Among many sample matrix components in urine, only a few are shown in the electropherogram. The first peak (denoted by an asterisk) is related to creatinine, identified by sample spiking. Creatinine, having pKa values of 4.9 and 9.2 and a structure similar to muscimol, was also effectively extracted from the sample (donor phase) into octanol, and then re-extracted to the acceptor phase.

The obtained enrichment factors were over 120 for MUS and 160 for PSC, respectively, i.e., recovery values of over 67% for muscimol and almost 90 % for psilocin. The preconcentration effect of the analyzed spiked urine samples was only 10–15 % lower compared with the solution of standards prepared in distilled water. The calibration curves of the studied analytes in spiked urine samples were linear in the range of 0.05–50 mg L^−1^, with the correlation coefficients from 0.9911 to 0.9992. The limit of detection in urine was 0.004 mg L^−1^ for PSC and 0.016 mg L^−1^ for MUS at a signal-to-noise (S/N) ratio of 3. The limits of quantification varied from 0.014 mg L^−1^ for PSC and 0.045 mg L^−1^ for MUS. The intraday and interday relative standard deviations (RSD, n = 6) of migration time (n = 6) were in the range of 0.3–8.5% and 1.0–11.3%, respectively.

## 3. Materials and Methods

### 3.1. Chemicals

**Figure 5:**
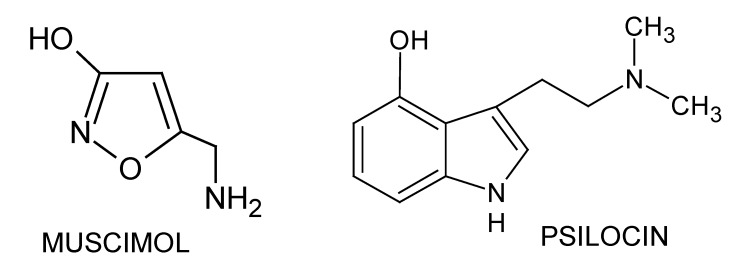
Chemical structures of muscimol and psilocin.

Muscimol (pKa: 4.8 and 8.4 [1,43]) was purchased from Abcam (Cambridge, UK). Psilocin (pKa 9.41 [4]) was from Sigma-Aldrich (Poznań, Poland). The chemical structures of the investigated analytes are shown in Figure 5.

A stock solution of the studied analytes was prepared in methanol (100 µg/mL) and stored at 4 °C. Sodium phosphate heptahydrate, toluene, dichloromethane, ethyl acetate, and octadecyltrimethoxysilane (ODTS) were obtained from Sigma-Aldrich (Poznań, Poland). Orthophosphoric acid (85%), octanol, and sodium hydroxide were purchased from POCH. S.A. (Gliwice, Poland). Deionized water was obtained from the Milli-Q system (Millipore, MA, USA).

### 3.2. SDME–CE Procedures

**Figure 6:**
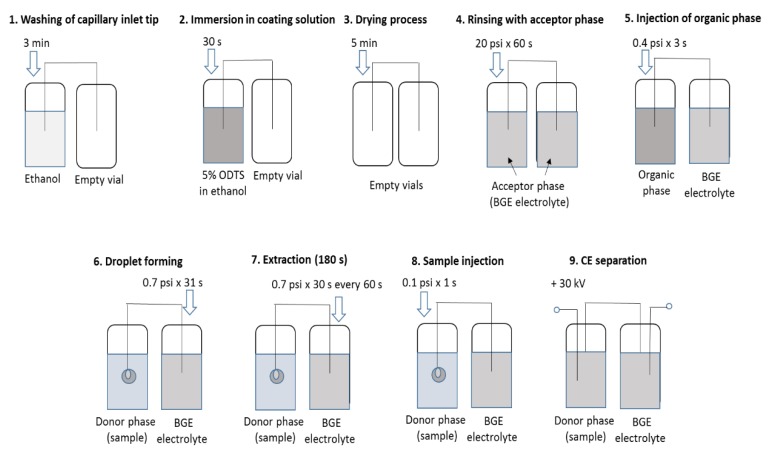
Scheme of SDME-CE procedure.

All SDME–C.E. measurements were performed on a PA 800 plus Capillary Electrophoresis System from Beckmann Coulter (Brea, CA, USA) equipped with a PDA detector. All analyses were undertaken at a wavelength of 214 nm. An uncoated fused-silica capillary 57 cm in length (50 cm to the detector) and with an inner diameter of 50 μm (Polymicro Technologies, Phoenix, AZ, USA) was used for SDME analysis. This gave an acceptor phase of 1.11 μL. The capillary was conditioned daily by washing it with 0.1 M NaOH (10 min), water (10 min), and BGE electrolyte (25 mM phosphate buffer pH 3, for 5 min). The voltage applied for the C.E. separation was held at 30 kV during analysis. The temperature was maintained at 18 °C. Before each run, the capillary inlet tip was washed with ethanol (3 min), immersed in a mixture of coating solution consisting of 5% ODTS in ethanol (30 s), and dried in an empty vial for 5 min. This process increased the hydrophobicity of the capillary inlet tip and ensured the stability of the drop suspended at the end. Next, the capillary was filled with a running buffer (acceptor phase) for 60 s (20 psi), and then octanol (organic phase) was injected into it (3 s at 0.4 psi). Finally, the capillary inlet tip was transferred into a vial with a sample solution (donor phase), and backpressure was applied (31 s, 0.7 psi) to form a drop of acceptor phase covered with octanol. Then, the capillary outlet tip was transferred to the acceptor phase. The use of backpressure (0.7 psi) for 30 s every 60 s of the extraction allowed us to stabilize the drop size and shape. After 180 s of extraction, the enriched acceptor phase was injected into the capillary for 1 s at 0.1 psi. Next, the capillary inlet tip was placed in ethanol to remove octanol from the tip for 30 s, the capillary was placed in vials with the BGE electrolyte, and the electrophoresis process was conducted. The enrichment factor (EF) in the case of this Three-Phase SDME-CE system can be approximated as the ratio of final and initial concentrations of the analytes in the aqueous back-extractive and aqueous sample phases, respectively [30,44]. Extraction efficiency was calculated from the following equation: E = n_A_/n_I_, where n_I_ and n_A_ are the number of moles (analyte) input in the system during the extraction time and collected in the acceptor, respectively. E is an analytically important parameter and should not be confused with recovery. It can be measured directly (as E) or according to the alternative formula E’ = (n_I_ − n_w_)/n_I_, where n_w_ is the number of moles leaving in the donor phase after extraction. The value of E indicates how much of the input of analytes arrives in the acceptor, while E’ measures how much is removed from the donor into the organic phase. The scheme of the SDME procedure is described in Figure 6.

### 3.3. Urine Samples Analysis

Blank urine samples were provided by a healthy female volunteer who did not take any medication during the study. All urine samples were collected and stored in PTFE flasks at 4 °C before use. Before analysis, samples were spiked with psilocin and muscimol at an appropriate concentration level, diluted with water (1:1, *v*/*v*), adjusted to pH 4 (85% orthophosphoric acid), and filtered with 0.45 µm PTFE filters.

## 4. Conclusions

The purpose of this study was to develop a new, fast, and reliable SDME-CE method for the isolation, preconcentration, and determination of fungal hallucinogenic metabolites in urine samples. For the first time, the two different fungal derivatives, muscimol and psilocin (isoxazole and indole compounds) were analyzed in a biological fluid, i.e., human urine. It was demonstrated that the developed SDME-CE method provides not only the preconcentration of the studied analytes, but also an efficient sample clean-up step. The time-consuming sample preparation procedure has been practically omitted. The obtained LOD values are sufficient for qualitative and quantitative determination, and the method can easily be applied in analyses of urine samples during routine analytical procedures.
